# Parental willingness to pay for child safety seats in Mashad, Iran

**DOI:** 10.1186/1471-2458-11-281

**Published:** 2011-05-08

**Authors:** Lida Jarahi, Mojgan Karbakhsh, Arash Rashidian

**Affiliations:** 1 Department of Community Medicine, Mashad University of Medical Sciences, Mashad, Iran; 2 Sina Trauma Research Center, Tehran University of Medical Sciences, Tehran, Iran; 3 Department of Community Medicine, Tehran University of Medical Sciences, Tehran, Iran; 4 Department of Health Management and Economics, School of Public Health/Knowledge Utilization Research Center, Tehran University of Medical Sciences, Tehran, Iran

## Abstract

**Background:**

Iran has one of the highest rates of road traffic crash death rates throughout the world and road traffic injuries are the leading cause of years of life lost in the country. Using child car safety seats is not mandatory by law in Iran. The purpose of this research was to determine the parental willingness to pay (WTP) for child restraints in Mashad, the second most populated city in Iran with one of the highest rates of road traffic-related deaths.

**Methods:**

We surveyed 590 car-owner parents of kindergarten children who were willing to participate in the study in the year 2009. We asked them about the maximum amount of money they were willing to pay for car safety seats using contingent valuation method.

**Results:**

The mean age of children was 33.5 months. The median parental WTP for CSS was about $15. Considering the real price of CSSs in Iran, only 12 percent of responders could be categorized as being willing to pay for it. Family income level was the main predictor of being willing to pay.

**Conclusions:**

The median parental WTP was much lower than the actual price of the safety seats, and those who were of lower socio-economic class were less willing to pay. Interventions to increase low-income families' access to child safety seats such as providing free of charge or subsidized seats, renting or health insurance coverage should be considered.

## Background

Iran has one of the highest death rates of road traffic crashes (RTCs) throughout the world (44 per 100,000) [1,2]. Iran's burden of disease study in 2002 identified injuries as the first cause of years of life lost in all ages and both genders (28% of the total) [3]. In 2000, around 423 under five children per 100,000 lost their lives due to RTCs [4]. RTC injury rate in Iran increased from 110 to 401 per 100,000 population during 1997-2005 due to rapid motorization and lack of strict car safety standards with a slight but significant decrease in 2006[5,1].

Child car safety seats (CSS) are effective in protecting children aged 0-4 years, if correctly used, and can reduce the risk of death by approximately 70% for infants and by half for toddlers [6,7]. While several countries have regulations for CSS use, its use is not necessitated by law in Iran. Thus, child safety seat utilization in Iran depends on the parents' decision making and choices. This is one of the reasons that highlights the importance of studying willingness to pay for child safety seat in Iran.

Determining the value that parents place on child injury prevention interventions can facilitate decisions for policy makers [8]. One approach is to study willingness to pay (WTP). In this approach, the maximum amount of money a person is willing to pay for a service is used for estimating the value of that intervention from the person's perspective [9]. One method for eliciting WTP is contingent valuation method in which a scenario on a specific commodity in a hypothetical market is used. It is a valid method for estimating the value placed by people on health care interventions [10,11]. Example of its used include assessing WTP for improved quality of care [12], in vitro fertilization [13] and reduction of mortality after myocardial infarction [14]. WTP has also been used in the areas of health promotion like fortification of foodstuffs [15], and disease prevention like prevention of cancer [16] and SARS [10]. Meanwhile, there are few studies assessing WTP for valuing someone else's health state (e.g. parental WTP) [8,9,17,18].

The objective of this paper was to assess parental willingness to pay for child car safety seats in Mashad, Iran.

## Methods

This cross-sectional study was conducted in Mashad in July to October 2009. Mashad is the second largest city in Iran with a population of about 3 million at the time of study [19]. In Mashad, the death rate of RTCs is high(46.4 per 100,000 population including all age groups[4]).

Participants were parents of kindergarten children who owned personal automobiles and were willing to participate in the study. We aimed for a sample of 590 parents considering a 1.5 design effect. In the pilot, 49 percent of parents were willing to pay the amount of money which was nearly adequate for buying a CSS in Iran (100$ or above). Multistage cluster sampling was used for selecting participants in which Mashad districts served us as strata and kindergartens as clusters. From each of 10 municipality districts of Mashad, 5 kindergartens were selected randomly for data collection (50 kindergartens in total). From each kindergarten, all parents who met the inclusion criteria (parents of children aged less than five years, who owned cars) were invited to participate in the survey. For each child we interviewed only one parent, as no significant differences were observed between the reported WTP by the mother and father of each child in the pilot study.

Interviews were performed in selected kindergartens using an interviewer-based questionnaire. The questionnaire included demographic data, parent's perceived efficacy of child safety seat and whether the parent had previously received any advice on using the safety seats. Past history of RTCs in the family was also recorded. Then, the parents were provided with pictorial introduction of the main types of safety seats which are recommendable according to the child's age. It included the presentation of a safety seat scenario including information on child car passenger deaths in Iran and available evidence on efficacy of safety seats. Then they were asked to choose from the available options the maximum amount of money they were willing to pay for having a seat. Contingent valuation was used with multiple choice questions for assessing WTP among parents. These options ranged from zero to $300 in seven choices that were developed based on the pilot study.

The median and mean amount of money the subjects were willing to pay was estimated, and the relationships with the parents' and children's characteristics were assessed. Kruskal-Wallis and Mann-Whitney U tests were employed to study the WTP value according to the study variables using SPSS software for Windows 15.0. WTP was also transformed into a dichotomous categorical variable. This variable was coded zero for WTP less than the minimum amount of money enough to obtain a CSS in Iran(<$100) and 1 for WTP enough to buy a CSS(≥$100). Logistic regression was used to develop the econometric method. A P value less than 0.05 was considered statistically significant.

### Ethical Approval

The proposal of this research was approved by the ethical committee of Sina Trauma Research Center affiliated to Tehran University of Medical Sciences. Our research conformed to the Helsinki Declaration and local legislations on Ethical Principles for Medical Research Involving Human Subjects. Each potential subject (parent) was adequately informed of the aims, methods, sources of funding, institutional affiliations of the researchers and the anticipated benefits of this research for the society. He/She was also informed of the right to refuse to participate in the study. The interview was performed after ensuring that the parent has understood the information and is willing to participate.

## Results

A total of 590 parents who met the inclusion criteria and were willing to participate in the study were included. The included parents reported the mean age of their children to be 33.5 months (SD = 16). Demographic characteristics of the responders and their children appear in Table 1.

**Table 1:** Demographic characteristics of study participants

Characteristic		Number	Percent
**Child Age groups(months)**	0-11	69	11.7

	12-23	126	21.6

	24-35	98	16.6

	36-47	163	27.6

	48-59	134	22.7

**Sex**	female	303	51

	male	287	49

**Mother's level of education**	(university education)	286	49

**Father's level of education**	(university education)	312	53

**Household income group**	Low income	<$500	282 (47.5)

	Moderate income	$500-$1000	195 (33)

	High income	>$1000	53 (9.5)

		Didn't answer	60 (10)

	**Total**	**590**	**100**

Of parents, 181 (31%) had previously received some advice on child safety seat use. Forty three parents (24%) said that they had heard about the seats from the media (newspapers, radio or television), 119 (66%) had heard from relatives or friends, and seven (4%) from their physicians. In addition, 117 (20%) parents reported previous experience of RTCs in their families, and in 21 per cent of those, a child passenger had been injured.

Of the 590 study participants, 13 (2.2%) were not willing to own a safety seat, even if it were free. The median parental WTP in participants who wanted to have safety seats (n = 577) was $15 (according to 2009 monetary values) with inter-quartile range of $15 to $45 and mean of $52 (SD = 66). Eighty two parents (14%) were willing to have safety seats, only if it were free of charge. Exclusion of these parents raised the median of WTP to $45 and mean to $60 (SD = 68)(Table 2).

**Table 2:** Amount of parental WTP for safety car seat in the study population according to the child's age

Amount of WTP	Families with an Infant	Families with a 1-5 year-old child	Total
	N (%)	N (%)	
Free(zero)	1 (1.5)	81 (16)	82 (14)

$15	39 (57.5)	180 (35)	219 (38)

$45	10 (15)	127 (25)	137 (24)

$80	8 (12)	60 (12)	68 (12)

$120	0 (0)	16 (3)	16 (3)

$180	0 (0)	11 (2)	11 (2)

$250	10 (15)	34 (7)	44 (7)

**Total**	69 (100)	383 (100)	**577 (100)**

Of responders (n = 590), 53 (9%) believed that preventive effect of safety seats in RTCs to be nil or trifle. Respondents in this category were more likely to report low (<$500/mo) to moderate ($500-$1000/mo) household income (P = 0.01). All 69 parents who had no information about safety seats had low economic and low educational status (P = 0.01). Of the respondents who underestimated the efficacy of safety seats in RTCs prevention, only six percent were willing to pay higher than $100. In addition, among parents without previous knowledge of safety seats, no one was willing to pay higher than $100 and about half of them indicated zero valuation.

Median WTP in higher economic status families were three times of that reported by parents from low income households. Similar to parents from low income (<$500/mo) households, those with low educational status were also associated with lower WTP (P < 0.001).

In general, higher household income status and higher parental educational level were associated significantly with the amount of money they were willing to pay (P < 0.001). Age and gender of the child, receiving previous advice about safety seats, past history of RTCs and having another under five child were not associated with parental WTP. In addition, there were no statistical differences between male and female respondents (P = 0.69).

WTP was also treated as a categorical variable with 85.8 percent of the study population(n = 506) not being willing to pay enough for a CSS(considering the real price in the Iranian market), 12 percent being willing to pay(n = 71) and 2.2 percent(n = 13)missing i.e. not willing to have the seat, even if it were free. Income group of the household (P < 0.001), educational level of mother(P = 0.001) and father(P = 0.006) and perceived efficacy of CSS in reducing death due to RTCS(P < 0.001) were significantly associated with being/not being willing to pay. In logistic regression, the only variable which remained as the independent predictor was household income status. Thus, the econometric model of WTP for CSS can be formulated as:

Logit (probability of being willing to pay for a CSS) = -5+1.36 household income group (categorized as 0 with monthly family income <$500; 1 with monthly family income ≥$500 and < $1000; and 2 if ≥ $1000).

Table 3 shows the details of logistic regression model with odds ratios, confidence intervals and significance levels.

**Table 3:** Relationship of parents' characteristics with their being/not being willing to pay for child safety seats (CSS) in logistic regression model

Variable	Categories	Number of Cases	Odds Ratio	0.95 Confidence Interval	P value
Household Income group	Low	274	1(baseline)	-	< 0.001٭

	Moderate	193	5.336	2.33-12.23	< 0.001٭

	High	51	16.26	5.81-45.46	< 0.001٭

	Negative	39	1(baseline)	0.25-4.33	0.016٭

Belief in efficacy of CSS in reducing fatal traffic injuries	To some extent	147	1.04	0.25-4.33	0.96

	Positive	278	3.21	0.89-11.53	0.07

	Don't know	54	0	-	0.98

Mother's educational level	Lower than high school certificate	62	1(baseline)	-	0.84

	High school certificate(diploma)	191	0.9	0.24-3.38	0.87

	Bachelor (BS/BA)	240	1.3	0.33-5.17	0.71

	Master(MS/MA) or above	25	1.08	0.19-6.17	0.93

Father's educational level	Lower than high school certificate	70	1(baseline)	-	0.57

	High school certificate(diploma)	163	0.18	0.05-0.63	0.007٭

	Bachelor (BS/BA)	240	0.34	0.1-1.41	0.08

	Master(MS/MA) or above	45	0.27	0.06-1.21	0.088

٭ Statistically significant

## Discussion

This study represents the first attempt to investigate parental WTP for CSS in Iran. This study seems especially important in Iran where people have to pay out-of pocket for more than half of their health care costs [20].

Except for 2.2 percent of participants who were not interested in having and using CSS for their children, others were willing to have it. On the other hand, their WTP were much lower than the real price of the seats in the market which is about $100 in Iran. Even with exclusion of parents who were willing to have CSS only if it were free, median amount of WTP was much lower than the minimum price of CSS in Iran. Thus, interventions to increase access of low-income families to safety seat, for example providing free of charge or subsidized seats, renting or health insurance coverage should be considered as options. In a study using WTP to assess public preferences towards fortification of foodstuffs with folic acid, 11 percent were willing to pay, 16% had zero WTP and 51% wanted their insurance to pay for it [15].

Nine per cent of the respondents estimated the preventive effect of safety seats in RTCs to be nil or trifle. In this subgroup, the low perceived efficacy can at least partially explain their low WTP amount. In a research studying parental WTP for improving pedestrian safety, some parents did not want to participate in the program; because they did not believe that the program would be effective [8].

Our study showed that the amount of money that parents were willing to pay for car safety seats was associated with educational and economic status of the household. Other reports from Turkey and the US have also shown parents of higher socio-economic class to have higher WTP [18,8]. Using a contingent valuation questionnaire, Mataria et al, have also demonstrated that WTP for "luxury" quality attributes is negatively affected by both income-dependent and income-independent impoverishment [12].

One fifth of families reported past history of RTCs; but, we did not detect any significant relationships between this experience and their WTPs. Further research is necessary to explore the association of perceived risk of injury and WTP for safety seats.

In addition, WTP was not associated with the number of children in the family. This might have been at least partially due to the high prevalence of one-child families in our sample. There was no relationship between parental WTP and child gender. This is generally in line with gender equity in receiving health care services for children in Iran.

In our sample less than a third of parents had received any advice about use of safety seats. In a similar study in Turkey, 72% of parents were informed about the safety seats [18]. Moreover, the source of advice in our research was mostly from relatives and lay people, and then from the media, and only 4% reported receiving advice from physicians. Similarly in Turkey, the main source of parents' information about safety seats was relatives or friends and only 3% from physicians [18]. Public campaigns to increase knowledge of people about safety seats are necessary. Future research should elaborate knowledge and attitudes of family physicians and pediatricians about safety seat utilization.

There were potential sources of strategic, information and interviewer bias in our study. Strategic bias is when the participants guess that the results of the study are to be used for decision making and feel that it is in their interest to give a lower or higher valuation than the true value [21]. For reducing information and interviewer bias, we had set a fixed pictorial scenario, fixed scenario sentence, and pictures of safety seats in the questionnaire.

Moreover, there are differences between community and governmental perspectives in WTP for preventive interventions in comparison with curative programs. For instance, in the USA, the reported WTP for care was three times higher than WTP for preventive intervention regarding one output [22]. This fact can be accounted as a limitation in comparison of our results with findings of published papers on WTP for services of non-preventive nature. On the other hand, published papers using WTP methods in the field of injury prevention are rare; except for two published papers on WTP on car crash protection [23], pedestrians' safety [8] and one on child safety seats[18].

In studying willingness to pay, payment cards are frequently used for contingent valuation, in which the respondents select their choice from cards or options ranging from zero to a pre-determined value [24-26]. In our study we used the multiple choice method instead of payment cards, as in the pilot study, it seemed to be more understandable and acceptable to our respondents. Previous studies comparing dichotomous choice and payment card approaches have identified no evidence of range bias or mid-point bias [26]. Nevertheless, multiple choice questions might not be the best method for eliciting WTP. Other methods like conjoint analysis or discrete choice modeling have been introduced [27]. Further research is necessary to demonstrate if feasibility of application, justifies its general application instead of payment card.

One of the difficulties with CVM studies is making the scenario realistic to the respondent [11]. For this purpose, the questionnaire of this research was only offered to parents who had a car in their family. In addition, they were provided with short information on the toll of road traffic deaths among pediatric car passengers in Iran and a pictorial introduction about CSS. This inclusion criterion could potentially limit the generalizability of our findings as car-owned families might be of higher economic status and thus have higher WTP. Despite this fact, more than 80 percent of these families were in low-middle income groups, minimizing the real potential for this bias.

## Conclusion

The main finding of this research was low level of parental willingness to pay for child car safety seats against moderate to high perceived efficacy. The main predictor for willingness to pay was family income level. A considerable unmet need was also observed regarding CSS advice from the physician responsible for the care of the child/family. This research can also be of practical importance for health policy makers to initiate CSS insurance coverage or lending programs parallel to decisions for obligatory CSS regulations. Future research can examine the effectiveness and feasibility of methods suggested in the paper to promote CSS use in Iran.

## Competing interests

The authors declare that they have no competing interests.

## Authors' contributions

MK proposed the idea of this research and participated in the proposal development, data analysis, interpretation and development of the manuscript. LJ participated in the proposal development, data collection and analysis, interpretation and development of the manuscript. AR participated in the proposal development, interpretation of willingness to pay findings and development of the manuscript. All authors have read and approved the final manuscript.

## Pre-publication history

The pre-publication history for this paper can be accessed here:

http://www.biomedcentral.com/1471-2458/11/281/prepub
